# The New York Institute of Stomatology

**Published:** 1901-02

**Authors:** 

**Affiliations:** The New York Institute of Stomatology


### Reports of Society Meetings.

### THE NEW YORK INSTITUTE OF STOMATOLOGY.

A REGULAR meeting of the Institute was held on Friday evening, November 9, 1900, at the residence of Dr. T. W. Onderdonk, No. 838 President Street, Brooklyn, N. Y., Dr. S. E. Davenport, of the Executive Committee, in the chair.

The minutes of the last meeting were read and approved.

### COMMUNICATIONS ON THEORY AND PRACTICE.

The secretary read the following extract from a letter which had been received from Dr. George H. Maxfield, of Holyoke, Mass., together with two specimens which were passed around. The specimens consisted of fillings composed of tin, on the surface of which gold had been burnished by means of ivory points.

"Last week we had a very profitable and enjoyable meeting at Providence. I think it one of the best we ever held in New England. The papers were of a high order, but as the programme was rather crowded there was not much discussion.

"I suppose you have heard of the clinic or demonstration that Dr. T. D. Shumway, of Plymouth, Mass., gave us at Springfield at our September meeting, of his method of combining gold and tin. We had him at Providence, and it created as much interest as anything we had there. I think it would well repay you to get him for one of your afternoon meetings. He tells me he has not demonstrated this since he has perfected the method, and that he would like the opportunity of coming before the Institute. I think it one of the best things brought out in dentistry for many years. Since he was at Springfield I have put in quite a number of these fillings.

"Dr. Stockwell says it has given him more enthusiasm than anything that has been brought out for a long time. He thinks it the most scientific and therapeutic of anything we have.

"I enclose a tooth which Dr. Shumway was showing at Boston. This cavity is filled almost to the edge with tin, and then the gold is burnished right on to the tin.

"I have filled a number of proximal cavities in incisors in this way. It is surprising what an affinity gold and tin have for each other. To demonstrate this I have a piece of pure tin about as big around as a cent and one-quarter of an inch thick. I filed a flat place in the edge and burnished on gold beyond the circumference, and then polished as in a tooth. Then on the flat side I burnished on quite a little knob of gold. All this burnishing was done with ivory points.

"As we know, tin is more compatible to tooth-structure than either cohesive gold, cement, or amalgam. By this method we get the therapeutic benefit of the tin and the beauty and wear of the gold. It is also much easier put in for both patient and operator. The ease with which it is done will surprise you. This method of packing the tin is quite an improvement on any other. It is surprising how solidly this is done by the use of hot instruments.

"If you do get Dr. Shumway, I am certain you will agree with me that it is one of the best things brought out since cohesive gold."

*The Chairman.*—Dr. Shumway's skill in the use of ivory points is known to be very great. These specimens are interesting in that they show the great affinity which the two metals have for each other.

*Dr. G. A. Wilson.*—Do we understand these fillings to be a combination of gold and tin?

*The Chairman.*—They are tin fillings over which gold has been burnished. They differ from the ordinary combination gold and tin fillings in that the gold and tin are not folded together before the material is inserted, but the pure tin is inserted first and the gold burnished on afterwards. If there is no further discussion upon this point we will listen to a short paper entitled "The Use of an Assistant," by Dr. Albert H. Brockway.

(For Dr. Brockway's paper, see page 75.)

*The Chairman.*—We have listened with great pleasure to Dr. Brockway's poem, and would be much pleased to have other stanzas added.

*Dr. J. Morgan Howe.*—It is always a great pleasure to hear Dr. Brockway, and it has been especially so this evening. His paper contains very valuable suggestions, and I wish to endorse what he has said about the advantage of having an assistant at the chair. I have had my own capacity considerably increased by such assistance, and in numerous ways my labors have been lightened. I would suggest, however, to those contemplating the adoption of that plan, and even to some who are accustomed to such help, that the presence of a third person looking at their dental deficiencies is objectionable to some patients. A minority of my patients, but still quite a number have shown a dislike to having an assistant near, and I think it worth while to humor their sensitive feelings as much as I can without inconvenience. Only a few days ago I got a letter from a gentleman asking for an appointment for the examination of his teeth, in which he wished it might be arranged so that there would be no observers of the conditions of his mouth excepting myself. I would also suggest that all practitioners should be alive to the feelings of their patients and not impose unpleasant conditions further than is required by the necessities of the case. Another suggestion is this, that one of the chief uses of an assistant should be the cleansing and sterilization of instruments. The sterilization of instruments is not so generally practised as it should be, and must be in future. The necessity of it may seem doubtful to many, because of the rarity of unfavorable results following its neglect, but the most remote chance of conveying disease germs from the mouth of one patient to another must be eliminated, or the dentist neglecting such safeguards will be liable to suffer.

*Dr. E. H. Raymond.*—I have been very much pleased with the paper that we have heard to-night. We might adopt many of the suggestions made by our friend, and would find that our work would be rendered much easier by the employment of a competent assistant. Dr. Brockway undoubtedly has more detail work for the assistant to do than most of us would require, but this is a matter of education and individual necessity. Too much importance cannot be placed on the subject of antisepsis and the sterilization of instruments. Carelessness in this direction may lead to serious consequences. An effectual method is to have a tumbler half-full of hydrogen dioxide on the operating-case, and immerse the instruments in that after using them; then have the assistant wash them with hot water and soap.

*Dr. J. A. Schmidt.*—I have listened with pleasure to Dr. Brockway's paper, inasmuch as it was through his influence that I was led to the adoption of an assistant. I find my assistant's time is largely taken up with the cleansing of instruments. They are made mechanically clean and then are sterilized in a formalin sterilizer. It was but yesterday that the importance of sterilizing instruments was brought home to me when I forced a pair of pliers into the palm of my hand. The instrument being sterile, I had no trouble with the wound. The assistant also does good work in assisting in examinations, marking down the cavities on the chart as I call them off. In malleting gold they are invaluable, also in treating teeth and preparing dressings. I am sure I have found them a good investment, and I thank Dr. Brockway heartily for suggesting the matter to me.

*The Chairman.*—I take pleasure, gentlemen, in introducing Dr. George S. Allan, who will describe and illustrate "Boxings."

(For Dr. Allan's paper, see page 73.)

*The Chairman.*—Do you take the impression of the cavity with a cup or simply with a pellet of compound?

*Dr. Allan.*—I use a little Russia-iron cup roughly modelled to cover the tooth-cavity, and when contouring is required the adjoining tooth is also included. I sometimes chill the impression-material and sometimes I do not. Very frequently at the lower or cervical margin of a cavity I place a layer of gutta-percha. Then taking my impression, after the box is made I leave this portion filled with gutta-percha. This vulnerable point is then protected by a stopping of gutta-percha, which, when it is worn out, can easily be replaced.

*Dr. Brockway.*—I would like to ask Dr. Allan if, in case one had the proper equipment, it would not be as easy to make a porcelain inlay as one of these boxings.

*The Chairman.*—The Executive Committee has endeavored to ascertain if this method of filling teeth which Dr. Allan terms "Boxings" is entirely new. With that idea in view they have written to Dr. Head, of Philadelphia, and Dr. Ames, of Chicago. The committee has received the following letter from Dr. Ames, together with a number of reprints of the article mentioned:

"CHICAGO, November 2, 1900.

"In reply to yours of October 17, I cannot do much beyond sending to you some reprints of a paper which I read before the Chicago Dental Society in December, 1898, since which time I have changed my methods very little. The description here would be as I would give it to-day, except that I would make mention of inlays, burnished shoes, or what your essayist, I fancy, terms boxings, for the support of a part or of parts of a bridge.

"I have often preserved the face of a tooth almost in its entirety while shaping the posterior surface for burnishing an attachment which will satisfactorily carry a single tooth to fill a space or to serve as one abutment of a bridge having two or more attachments. A compound cavity can often be filled with an inlay which will satisfactorily carry a dummy to fill an adjoining space.

"I would also call attention to a simpler method of obtaining contours of inlays in cases where the color of the surface of metal is not an important factor. With a nitrous oxide or oxygen blow-pipe, or any blow-pipe giving a fine pointed flame of intense heat, small pieces or globules of refractory metal (alloyed gold) can be added to the surface of the nearly completed inlay at the points which need extension, solder being used between and over these particles.

"I will only add that these operations continue to give me much satisfaction. I trust that in your discussion there will not arise the usual confusion of this solid gold inlay, with which the matrix is burnished into the cavity, and the shell affair which I have heard credited to Dr. Bing, of Paris, which, I should say, does not, from the nature of the procedure, give as promising a joint. I have several times heard gentlemen denounce gold inlays as untrustworthy, and immediately tell of having cemented gold-foil fillings into the cavity from which they had loosened from want of sufficient anchorage, with most satisfactory results, which simply proves that the secret of a trustworthy inlay is simply a proper plan and execution.

"Trusting that this will reach you in good season, I am,

"Yours truly,

"W. V. B. Ames."

*Dr. Parker.*—Fifteen or sixteen years ago I read a paper explaining a facing which, from the specimens shown, I should judge was identical with those of Dr. Allan.

*The Chairman.*—I think Dr. Parker is mistaken in thinking Dr. Allan's boxings are essentially facings; they differ materially from those described by Dr. Parker.

*Dr. Raymond.*—We are all under obligations to Dr. Allan for presenting to us such a unique method of reconstructing broken or decayed teeth. When I read the announcement of this meeting, and saw that Dr. Allan would describe "boxings," I was rather puzzled, not having heard that term in connection with dentistry. I certainly had no conception of the beautiful work which has been presented to us this evening, and did not realize that such results could be obtained by the use of gold plate. Although this is the porcelain era, it is gratifying to know that the gold standard is still in operation in this country. But while the results are so beautiful, from the doctor's description, it seems to me that such results cannot be obtained except by a process which is somewhat complicated and which would consume more time than most of us could well give to it. Will Dr. Allan inform us whom he employs to assist him?

*Dr. Allan.*—Dr. Leeming.

*Dr. Raymond.*—If we all could have the assistance of as skilful a man we might save our patients much discomfort by adopting this method, as it meets the requirements of a solid gold filling and the advantage of the non-metallic substance coming in contact with the tooth structure, in the cement which holds the "boxing" in place. A simple and quick method of filling a large cavity in a frail tooth with pure gold plate is as follows: after preparing and shaping the cavity and bevelling the edges, take a piece of sheet tin about 26 gauge and restore the contour of the decayed tooth as accurately as possible. Take this tin pattern, and after flattening it out duplicate in pure gold by placing it on the plate and marking its outline with a sharp point. Cut this out and with pliers and burnishers fit it to the cavity as was done with the tin, then solder one or more anchors on the inside of the cap (which it now becomes) and cement it in place with zinc phosphate. In attaching the anchors use 14-carat solder, which flows by holding over a spirit-lamp. The "boxings" are in every sense more artistic, but when pressed for time this latter plan will be found to give gratifying results. I congratulate the doctor on his new method and the Society on having the benefit of it.

*Dr. Howe.*—I would like to ask Dr. Allan whether these boxes which he makes are soldered together all around the margins so as to be air-tight when completed. I take it from what he has said that this is the case. If so, it is possible, having cut his holes in the bottom of the box, to fill it solid full of cement. It seems to me that it is similar to various methods of treating root-canals which have been described, of squirting the medicine into the canal without considering what is to become of the air. It occurred to me that the air would prevent the cement entering the box.

*Dr. F. Milton Smith.*—I have been very much interested in Dr. Allan's description, and am delighted with the beauty and the workmanship of these boxes. As to the result obtained, I do not see that it is so different from that obtained in other ways. The principal thing which attracts me is that so much of the work can be done by one whose time is not so valuable as that of the "successful dentist." If it is possible that we can turn over this work, that takes a long time to do, to a plate-worker, we have accomplished something. Dr. Allan is fortunate in having a man who can work to such fine lines. Dr. Allan has mentioned the inlays which were presented by Dr. Dwight Smith at the September meeting at

Dr. Davenport's. This work consisted simply in burnishing plate-gold into the cavity, forming a cup, and then filling with solder and grinding to get the contour. There is no doubt as to the durability of such inlays. The principal objection is the length of time required. There are several points connected with this work which go to make it successful, the principal one being perfect adaptation to the walls of the cavity. I think it would be very hard to find many plate-workers who would strike up from a die inlays which would be anywhere near perfect. These specimens are certainly most beautifully done. As to retaining them in place, the suggestion of making a hollow box in some respects is excellent. I hardly think Dr. Howe's objection would hold if the holes are sufficiently large. If necessary the box could be filled before it was set. I accomplish this in the solder inlay by drilling in from the under-side, making undercuts, and filling them with cement. The great point in all this work is that it shall absolutely fit.

*Dr. Charles O. Kimball.*—It seems to me that the suggestion of Dr. Allan is good and helpful. I can think of a great many cases in which it could be used to advantage. The method of making a gold cap described by Dr. Raymond is not like this of Dr. Allan. It seems to me that it has two marked points of usefulness. In the first place, it is tough as compared with a porcelain inlay. We all know of places upon which a great deal of the strain of mastication comes, and where we would hesitate to use porcelain because it is so brittle. A second point is that a boxing of this kind, filled as it should be, perfectly full of cement, would minimize the danger of the filling spreading under the strain of mastication. We must always remember, when we put in a crown filling which is intended to last for years, that the teeth are constantly wearing, and if we have a filling which will spread under the increased strain it may become imperfect and even split the tooth.

*Dr. L. C. Leroy.*—I should like to have Dr. Allan describe how he would prepare a filling of this kind for a cavity in a molar where there was a distinct undercut.

*The Chairman.*—It would seem possible, if the cement to be used in setting these boxes was of the slow-setting variety, that the interior of the box could be first filled with the cement through the holes referred to, and afterwards a portion of the cement be placed in the cavity and the box pressed to place. To the uninitiated this method of Dr. Allan's would seem to have a great advantage over the thin facings with the loop underneath, in that the strain of mastication coming on the facing is liable to cause the edges to turn up, giving an opportunity for the cement to wash out. The rounded edges of the boxings would certainly obviate that.

*Dr. Allan.*—As to Dr. Brockway's question, which is a very proper one, the box is superior to the inlay of porcelain, first, because of its greater strength, and secondly, because a better contour can be more easily obtained. Dr. Howe's question has already been satisfactorily answered. As Dr. Davenport suggests, the box is first filled with cement, an excess being placed on the under surface, then it is pressed into place. The suggestion has been made that it is very complicated and difficult work. Now, I have once or twice been caught without my plate-worker, and have made these boxes myself without any impression. I burnish in the under shell and fill or contour out with wax while the patient is in the chair; removing this I strike up the outer shell, then, placing the two together with some little pieces of solder around the margins, heat in the Bunsen flame till the solder flows, after which trim the edges and polish.

As to Dr. Leroy's question, I have already stated that the cavity must be prepared so that the impression can easily be withdrawn. This simply means to fill all undercuts with modelling compound or cement.

*The Chairman.*—Will Dr. Allan explain one point? In making the box which he has just explained, does he leave an excess of gold on the outer shell, which is trimmed off after it is soldered?

*Dr. Allan.*—That is exactly what is done. The boxing when completed should fit the cavity perfectly, as perfectly as a good filling when finished ought to. Much care should be taken in pressing the boxing in place. No hard substance should be used, or the surface may be badly indented. The finger alone should be employed, or the broad end of a soft stick with a bit of spunk interposed. A good plan is to partly fill the boxing with cement and allow it to thoroughly harden before setting with a fresh mixture. The make-up of the boxing must be varied to suit individual cases. Of course, in all cases it must be fashioned so as to easily fall into its place. While the outer shell must fit closely the margins of the cavity, the inner shell need not and oftentimes must not closely follow the outlines of the cavity. As an example, take a bicuspid having the mesial and distal faces destroyed and the two cavities meeting between the cusps. It is not difficult to so fashion the inner shell that without loss of strength to the whole it will when finished readily drop into place.

The annual meeting of the Institute was held on Tuesday evening, December 4, at the office of Dr. S. E. Davenport, 51 West Forty-seventh Street. The following officers were elected for 1901:

President, Dr. J. Morgan Howe; Vice-President, Dr. A. H. Brockway; Recording Secretary, Dr. F. Milton Smith; Corresponding Secretary, Dr. George A. Wilson; Treasurer, Dr. J. Adams Bishop; Editor, Dr. F. L. Bogue; Curator, Dr. J. G. Palmer.

*Executive Committee.*—For three years, Dr. C. O. Kimball; for Dr. Howe's unexpired term of two years, Dr. C. F. Allan.

Adjourned.

FRED. L. BOGUE, M.D., D.D.S.,
*Editor The New York Institute of Stomatology.*

